# The molecular epidemiology of respiratory viruses associated with asthma attacks: A single-center observational study in Japan

**DOI:** 10.1097/MD.0000000000008204

**Published:** 2017-10-20

**Authors:** Takeshi Saraya, Hirokazu Kimura, Daisuke Kurai, Haruyuki Ishii, Hajime Takizawa

**Affiliations:** a Kyorin University School of Medicine, Department of Respiratory Medicine, Mitaka City; b Infectious Disease Surveillance Center, National Institute of Infectious Diseases, Tokyo, Japan.

**Keywords:** adult, asthma attack, molecular epidemiology, respiratory virus

## Abstract

Few reports have described the significance of viral respiratory infections (VRIs) in exacerbation of asthma in adult patients. The aim of this study was to elucidate the profiles of VRIs in adult patients with asthma along with their molecular epidemiology.

A cross-sectional observational study was conducted at Kyorin University Hospital from August 2012 to May 2015. To identify respiratory pathogens in inpatients and outpatients suffering from asthma attacks, RT-PCR/sequencing/phylogenetic analysis methods were applied alongside conventional microbiological methods. Phylogenetic and pairwise distance analyses of 10 viruses were performed.

A total of 106 asthma attack patients enrolled in this study in both inpatient (n = 49) and outpatient (n = 57) settings. The total 106 respiratory samples were obtained from nasopharyngeal swab (n = 68) or sputum (n = 38). Among these, patients with virus alone (n = 39), virus and bacterial (n = 5), and bacterial alone (n = 5) were identified. The ratio of virus-positive patients in inpatient or outpatient to the total cases were 31.1% (n = 33) and 10.4% (n = 11), respectively. The frequency of virus-positive patients was significantly higher in inpatients (75.3%, n = 33) than in outpatients (19.3%, n = 11). Major VRIs included human rhinovirus (HRV) (n = 24), human metapneumovirus (hMPV) (n = 9), influenza virus (Inf-V) (n = 8), and respiratory syncytial virus (RSV) (n = 3) infections with seasonal variations. HRV-A and HRV-C were the most commonly detected viruses, with wide genetic divergence on phylogenetic analysis.

Asthmatic exacerbations in adults are highly associated with VRIs such as HRV-A or HRV-C, hMPV, RSV, and Inf-V infections with seasonal variations and genetic divergence, but similar frequencies of VRIs occurred in asthma attack patients throughout the seasons.

## 1 Introduction

Risk factors relating to the development of asthma attacks are multiple and complex. The frequency of viral respiratory infections (VRIs) in asthma attack patients is now recognized as having a major impact on asthma pathogenesis in children.^[1]^ Kusel et al^[2]^ reported that VRIs caused by human rhinovirus (HRV) and respiratory syncytial virus (RSV) in the first year of life were strongly associated with the diagnosis of current asthma and persistent wheeze in 5-year-old children. Furthermore, Jackson et al^[3]^ demonstrated that children had an increased risk of asthma at 6 years of age if they experience wheezing in the first 3 years of life, with RSV [odds ratio (OR): 2.6], HRV (OR: 9.8), or both HRV and RSV (OR: 10). Regarding adult asthma patients, previous reports showed that respiratory tract infections associated with asthma exacerbation ranged from 10% to 21% to 45%,^[4–6]^ of which 60% are HRV.^[7]^ However, especially in Japan, few epidemiological data exist on the correlation between VRIs and asthma attacks in adult patients^[1,8]^ from studies conducted within the year.^[9]^

Moreover, Martin et al^[10]^ and Kraft et al^[11]^ described the correlation between *Mycoplasma pneumoniae* infection and asthma attacks in adult patients; however, the preliminary findings could not confirm the evidence of *M. pneumoniae* infection in those patients.^[8,12]^ Therefore, we prospectively studied adult patients with asthma attacks to clarify the role of VRIs and/or bacterial infection, including *M. pneumonia,* and their molecular epidemiology.

## 2 Methods

### 2.1 Patients and study design

In this cross-sectional observational study, we prospectively enrolled adult patients suffering from asthma attacks visiting Kyorin University Hospital (a 1100-bed tertiary center in Tokyo) in both inpatient and outpatient settings from August 2012 to May 2015.

### 2.2 Inclusion criteria

Eligible patients were aged over 18 years and had a clinical diagnosis of asthma in addition to one or more of the following characteristics: variability in peak expiratory flow of more than 20%; airway reversibility by inhaled β2 agonist; hyper-responsiveness to methacholine challenge; and recurrent dyspnea episodes with wheezing. Asthma attack patients were enrolled in this study if they had acute or subacute episodes of progressively worsening shortness of breath, cough, wheezing, and chest tightness, or some combination of these symptoms, characterized by decreases in expiratory air-flow and objective measures of lung functions according to the latest National Institute of Health (NIH) National Asthma Education and Prevention (NAEP) guidelines/Global Initiative for Asthma (GINA) guideline 2012 or satisfied the moderate to severe exacerbation of asthma based on the *American Thoracic Society* (ATS)/*European Respiratory Society* (ERS) statement.^[1]^

### 2.3 Exclusion criteria

We excluded the patients who had chronic obstructive lung disease, pneumonia, interstitial lung diseases, acute heart failure, and respiratory symptoms that were possibly due to infections in the last month.

### 2.4 Sample and clinical data collection

Respiratory samples included sputum or nasopharyngeal swab collected at inpatient admission or at the outpatient setting at the time patients were diagnosed of asthma attacks. Clinical data were also obtained at the same time. Respiratory samples for PCR-based detection of respiratory viruses, *M. pneumoniae*, and *Chlamydophila pneumoniae* were collected separately from those intended for bacterial cultures and were stored at -80°C until use. Gram stain was performed on a purulent portion of each sputum specimen and examined by trained personnel. Sputum samples were considered as good quality for evaluation if they were classified as Geckler 4 or 5. Positive bacterial culture was based on acceptable sputum samples with predominant species and compatible results from Gram staining.

### 2.5 RNA extraction, (RT)-PCR, and gene sequencing of the pathogens

Samples were centrifuged at 3000 g at 41°C for 30 minutes. Viral RNA and DNA were extracted from supernatants using the QIAamp Viral RNA Mini Kit (Qiagen, Valencia, CA). Reverse transcription was performed using PrimeScript RT reagent Kit (Takara Bio, Otsu, Japan), according to the manufacturer's instructions. We used reverse transcription polymerase chain reaction [(RT)-PCR] to try to detect both DNA and RNA viruses, including human metapneumovirus (hMPV), human rhinovirus (HRV), enterovirus, respiratory syncytial virus (RSV), influenza viruses A, B, and C (Inf-A, B, and C), human parainfluenza viruses, human coronavirus, adenovirus, cytomegalovirus, human parvovirus B19, varicella zoster virus, and human bocavirus together with *M. pneumoniae* and *C. pneumoniae* as previously described.^[13,14]^ We used specific primer sets for the amplifications of these viruses as we also previously described.^[13,14]^ PCR products were purified using MonoFas DNA Purification Kit I (GL Sciences Inc., Shinjuku, Tokyo, Japan). The purified products were sequenced with a BigDye Terminator v3.1 Cycle Sequencing Kit (Applied Biosystems, Foster City, CA) using the above primers. Sequence analysis was performed on an ABI 3130 Genetic Analyzer (Applied Biosystems). The nucleotide sequences thus obtained were given GenBank accession numbers from LC020476 to LC020488. 2.7.

### 2.6 Phylogenetic analyses by the neighbor-joining (NJ) method and genotyping of hMPV, RSV, and HRV

We performed phylogenetic analyses using Molecular Evolutionary Genetics Analysis (MEGA) software, version 5.0. Phylogenetic analysis of hMPV, RSV, and HRV was based on parts of the *F* gene (317 bp), *G* gene (240–312 bp on RSV-A, 234– 294 bp on RSV-B), and *VP4/VP2* coding region (390 bp), respectively.

The species of the viruses were estimated using the basic local alignment search tool (BLAST).^[14]^ Evolutionary distances were estimated using the Kimura 2-parameter method and phylogenetic trees were constructed using the neighbor-joining (NJ) method. Reliability of the trees was estimated using 1,000 bootstrap replications. We calculated pairwise distances (*p*-distance) of the respiratory viruses detected in this study, hMPV, RSV, Inf-V, and HRV, using MEGA software, version 5.0. Calculations were based on the nucleotide sequences of each virus.

### 2.7 Ethical approval

Samples were collected after written informed consent was obtained from the subjects or their legal representatives. The study protocol was approved by the Ethics Committee on Human Research of Kyorin University Hospital (H24-021) on July 31, 2012. The protocols were carried out in accordance with the approved guidelines.

### 2.8 Statistical analysis

Statistical comparisons of nonparametric data were performed using the Mann–Whitney test or Wilcoxon signed-rank test. Comparisons of categorical data were made using Pearson Chi-squared test. All tests were 2-sided. A value of *P* < .05 was considered statistically significant. Data were analyzed using IBM SPSS version 20.0 software for Windows (SPSS, Chicago, IL).

## 3 Results

### 3.1 Clinical characteristics of inpatient and outpatient asthma attack patients

We examined a total of 106 asthma attack patients in the study period, in both inpatient (n = 49) and outpatient (n = 57) settings. Among these, patients with virus alone (n = 39), virus and bacterial (n = 5), and bacterial alone (n = 5) were identified. The ratio of virus-positive patients in inpatient or outpatient to the total cases were 31.1% (n = 33) and 10.4% (n = 11), respectively. The frequency of virus-positive patients was significantly higher in the inpatient group (67.3%, n = 33) than in the outpatient group (19.3%, n = 11) (Table 1). Patient age, sex, and the proportion of smokers were similar in both groups. However, the proportion of patients with hypoxemia (SpO_2_ ≤88%), wheezes, and patients classified as severe or serious asthma attack according to Japanese guideline^[15]^ and/or ATS/ERS statement^[16]^ were significantly higher in the inpatient group than in the outpatient group (Table 1). Body temperature was not significantly different (*P* = .054) between inpatients [median 37.0°C, interquartile range (IQR) 36.6–37.9) and outpatients (median 36.7°C, IQR 36.4–37.1]. Interestingly, the systemic inflammatory markers, such as serum white blood cell (WBC) count and C-reactive protein (CRP), were also comparable in both groups.

**Table 1:**
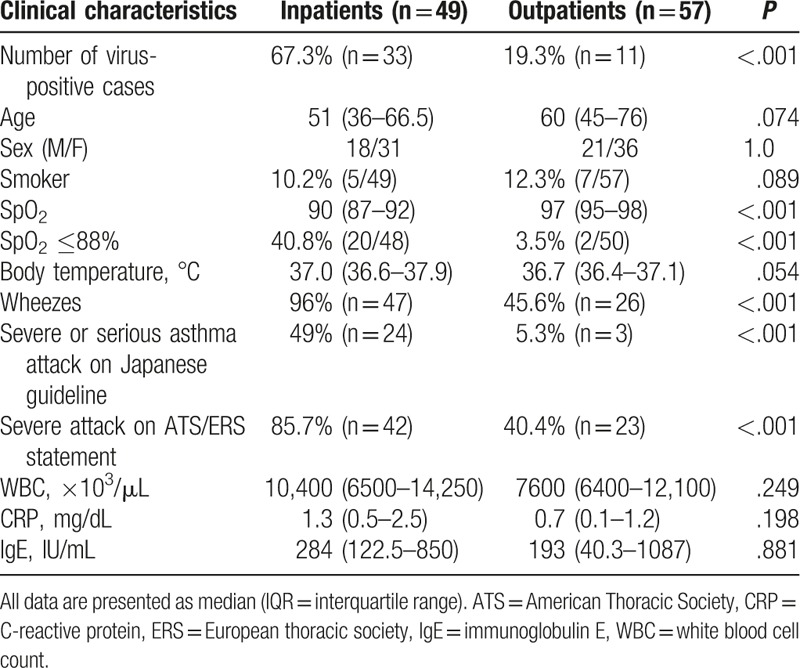
Comparison of the clinical characteristics of inpatient and outpatient asthma attack patients.

### 3.2 Clinical characterization of inpatients with or without virus infection

Of the 49 inpatients, 33 (67.3%) were virus positive (Table 2). The age and male to female ratio was comparable between virus-positive and virus-negative inpatients. Duration of asthma in years (median 25, IQR 20–45 vs median 10, IQR 5.2–22.5, *P* = .08) and the proportion of smokers (7.7% vs 37.5%, *P* = .051) were not significantly different in virus-positive and negative inpatients. In addition, the proportion of the patients with hypercapnia (PaCO_2_ ≥45 Torr) was not significantly higher in virus-positive inpatients than in virus-negative inpatients (39.1% vs 7.7%, *P* = .06).

**Table 2:**
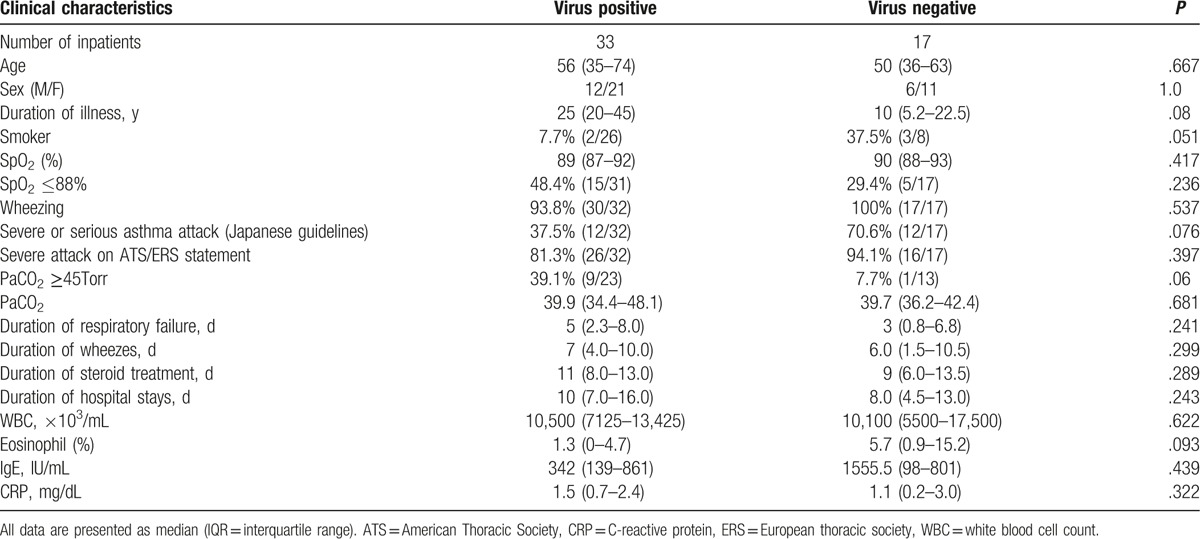
Clinical characteristics of virus-positive and negative inpatients.

Duration of respiratory failure (days), wheezes (days), steroid treatment (days), and hospital stays (days) did not differ significantly in virus-positive and negative inpatients (Table 2). Furthermore, the value of serum inflammatory markers on admission, such as WBC count and CRP, were equal in both groups.

### 3.3 Pathogen profile

The total 106 respiratory samples were obtained from the nasopharyngeal swab (n = 68) or sputum (n = 38). Among the 38 sputum samples, good quality samples (Geckler classification of 4 or 5) were only 19 samples, out of which 10 samples were positive for bacteria (Table 3).

**Table 3:**
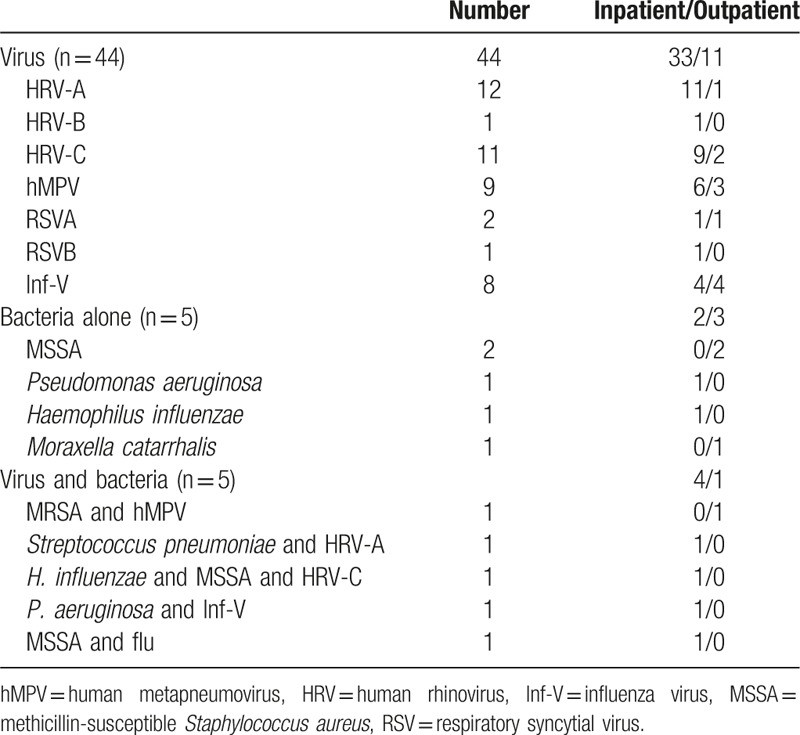
Detected pathogens.

Within the study period, we identified a total of 44 virus detection events obtained from the inpatient or outpatient settings. The virus detection event consisted of HRV (n = 24), hMPV (n = 9), Inf-V (n = 8), and RSV (n = 3) (Table 3). The main HRV species were identified as HRV-A (n = 12) and HRV-C (n = 11), with only 1 HRV-B infection. Among the virus-positive group (n = 44), the incidence of bacterial coinfection (bacteria plus virus) was 11.4% (n = 5), and the ratio of bacterial alone group (n = 5) to the total 49 pathogen positive patients was 10.2%, and no one had *M. pneumoniae* or *C. pneumoniae* infections.

### 3.4 Seasonal variations of detected viruses

**Figure 1:**
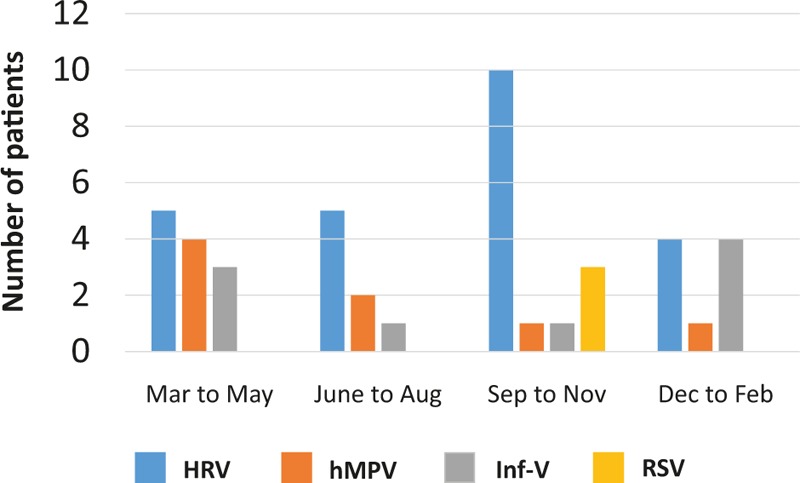
Seasonal variations of respiratory viruses associated with asthma attack patients. hMPV = human metapneumovirus, HRV = human rhinovirus, Inf-V = influenza virus, RSV = respiratory syncytial virus.

During the study period (approximately 3 years), respiratory virus infection was more common in the spring (March to May) and autumn (September to November) (Fig. 1). Of note, the frequency of HRV and RSV infection seemed to be high in autumn, while hMPV was likely to be indicated in spring. Similarly, Inf-V infection was most common in the winter and spring. The proportion of virus-positive patients to the total asthma attack subjects in each season was 38.7% (n = 12, spring), 50% (n = 8, summer), 53.6% (n = 15, autumn), and 32.2% (n = 9, winter). However, the incidence of virus infection in each season was not statistically significant.

### 3.5 Phylogenetic analyses by the neighbor-joining (NJ) method and genotyping of hMPV, RSV, Inf-V, and HRV

**Figure 2:**
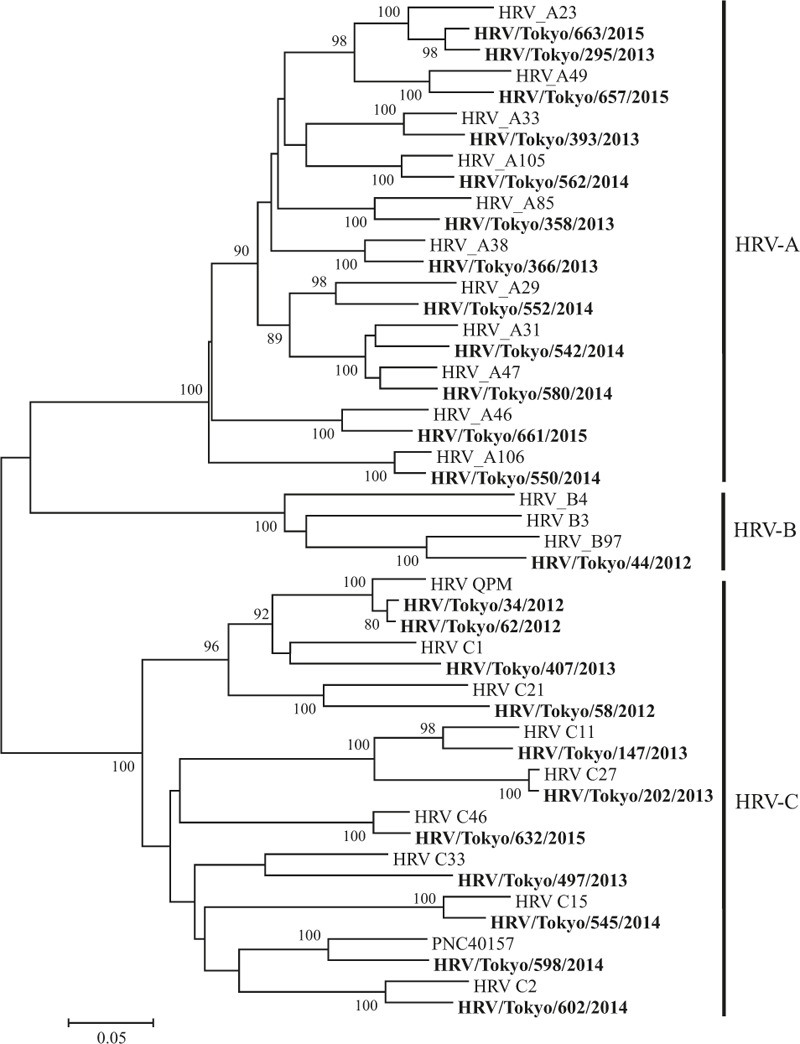
Phylogenic tree based on HRV *VP4/VP2* coding region.

**Figure 3:**
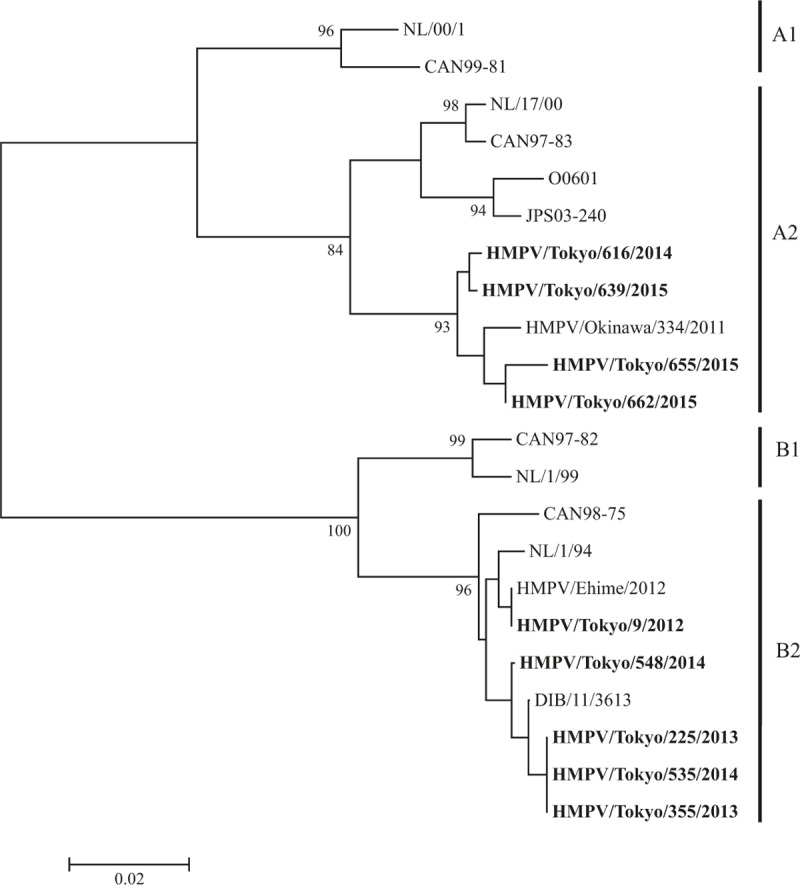
Phylogenetic tree based on hMPV *F* gene sequences.

**Figure 4:**
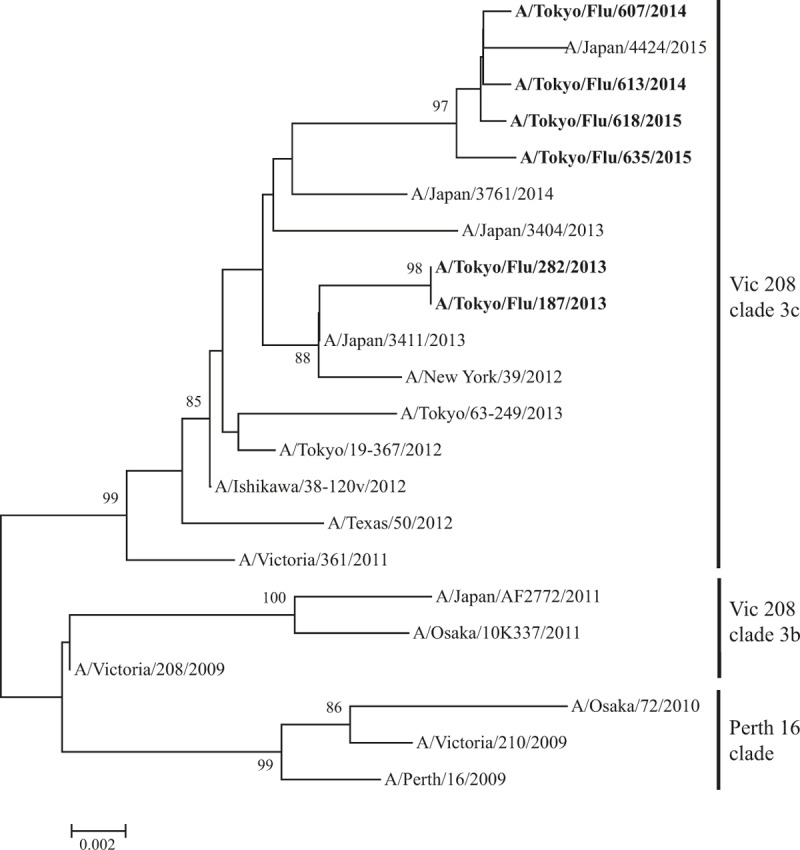
Phylogenetic tree based on InfV(H3N2) *HA1* gene sequences.

**Figure 5:**
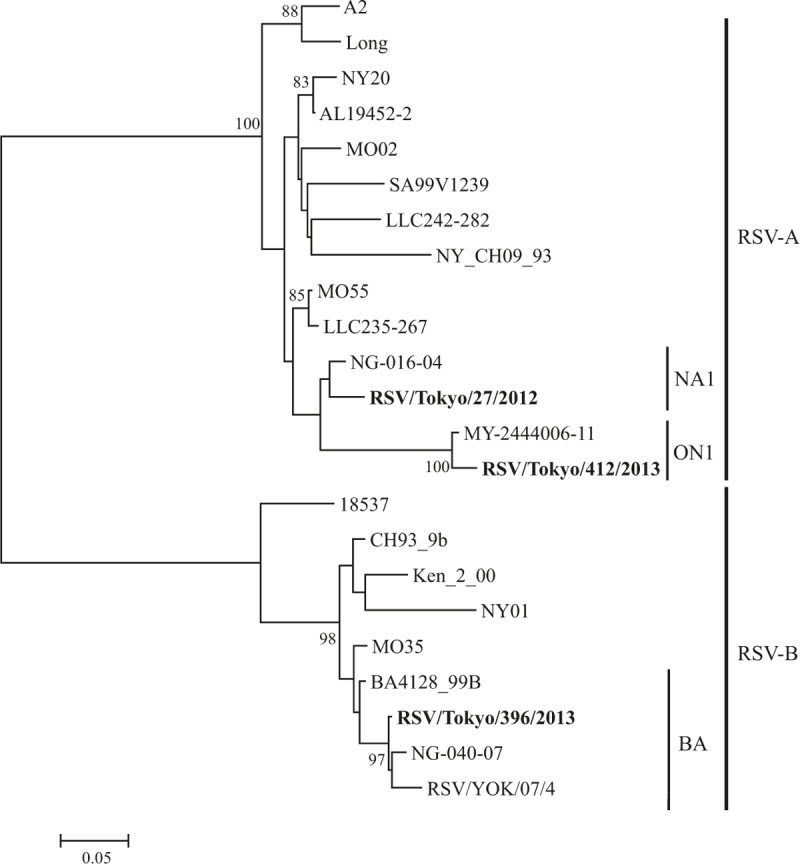
Phylogenetic tree based on RSV *G* gene sequences.

We performed phylogenetic analysis of HRV, hMPV, Inf-V, and RSV as a major cause of asthma attack in this study. HRV was the most commonly detected virus and consisted of diverse genotypes on phylogenetic analysis (Fig. 2). This trend was also found in the analyses of hMPV (Fig. 3), Inf-V (Fig. 4), and RSV (Fig. 5).

## 4 Discussion

This study was the first to show that various respiratory viruses such as HRV, hMPV, Inf-V, and RSV were associated with asthma attacks in Japanese adult patients.

HRV was the most common cause of asthma attacks in autumn, Inf-V was predominantly seen in winter to spring, and VRIs seemed to equally affect asthma attacks in every season in Japan during the 3-year study period. Moreover, the molecular epidemiological data suggested that the detected viruses had a wide genetic divergence (particularly HRV-A and HRV-C) with seasonal variations.

Fujitsuka et al^[14]^ showed that a majority of Japanese children with acute wheezing illness during a 17-month period had an RSV, HRV, or RSV and HRV infections. They also found that RSV was dominantly detected in patients with no history of wheezing and/or asthma, while HRV was dominant in patients with a history of wheezing and/or asthma. Indeed, the present study demonstrates that HRV is an important cause of asthma attacks, even in adult patients.

The molecular epidemiology of each HRV species associated with asthma attacks may not be completely known, because the HRV-C was recently recovered or nonculturable.^[17]^ Miller et al^[18]^ found that HRV-C has a stronger association with virus-induced asthma than HRV-A and HRV-B in hospitalized children younger than 5 years with acute respiratory illness. However, the epidemiological data of HRV associated asthma attacks in adults were scarcely reported in Japan. In this regard, the present study showed that HRV-A or HRV-C has a potential role in asthma attacks among Japanese adult patients, with a wide genetic divergence. As shown in Fig. 1, the detection of HRV was most common in autumn, indicating that the common cold, which results from HRV during the fall months, can lead to asthma attacks as reported in previous studies.^[19]^ Furthermore, previous reports described other respiratory viruses, such as hMPV,^[20]^ RSV, parainfluenza virus, Inf-V, and coronavirus, as a cause of asthma attack in adults,^[4]^ and the present study clearly demonstrated the main causative viruses are HRV, hMPV, RSV, and Inf-V.

What is more important is the finding in our study that the various VRIs can cause asthma attacks with a seasonal predilection in each virus; however, the frequency of VRIs in asthma attack patients seem to be equal throughout the seasons.

Interestingly, there was no infection or coinfection with *M. pneumoniae* or *C. pneumoniae*, which is contrary to the findings described in previous reports.^[10,11]^

Of note, regarding inpatients, virus infection seemed to be associated with hypercapnia (PaCO_2_ ≥45 Torr) and the duration of asthma (years), but the severity of wheezing and/or bronchial epithelial damage differed among respiratory viruses,^[4]^ thereby furthering the accumulation of index cases required to independently demonstrate the pathogenesis of asthma attacks or the clinical characteristics of each virus. The limitations of our study include lack of data for the severity of bronchoconstriction or inflammation in the lower respiratory tract and paucity of data about the different mechanisms of asthma attacks resulting from each virus infections, which might lead to the diverse respiratory symptoms or the development of airway remodeling.

However, the present study reports the first evidence of VRIs in asthmatic adult Japanese patients with a clear molecular epidemiology in a single-center cohort.

## 5 Conclusion

Asthmatic exacerbations in adults are highly associated with VRIs, such as HRV-A or HRV-C, hMPV, RSV, and Inf-V infections with seasonal variations and genetic divergence, but the frequency of VRIs in asthma attacks seemed to be equal throughout the seasons.
